# 5,6-Dihy­droxy-7,8-di­meth­oxy­flavone

**DOI:** 10.1107/S1600536813014451

**Published:** 2013-06-15

**Authors:** Lin-Lin Jing, Xiao-Fei Fan, Peng-Cheng Fan, Lei He, Zheng-Ping Jia

**Affiliations:** a Department of Pharmacy, Lanzhou General Hospital of PLA, Key laboratory of the prevention and cure, for the plateau environment damage PLA, 730050, Lanzhou Gansu, People’s Republic of China

## Abstract

The title compound (systematic name: 5,6-dihy­droxy-7,8-dimeth­oxy-2-phenyl­chromen-4-one), C_17_H_14_O_6_, is a flavone that was isolated from the petroleum ether-soluble fraction of the rare traditional Chinese medicinal herb *Saussurea involucrata*. The flavone mol­ecule is almost planar, with a dihedral angle between the planes of the benzo­pyran-4-one group and the attached phenyl group of 1.89 (6)°. The 5-hy­droxy group forms a strong intra­molecular hydrogen bond with the carbonyl group, resulting in a six-membered hydrogen-bonded ring. The 6-hy­droxy group also forms an intra­molecular O—H⋯O contact. In the crystal, the molecules are linked by O—H⋯O and C—H⋯O hydrogen bonds and π–π inter­actions [3.37 (2)–3.39 (2) Å], which build up a three–dimensional network.

## Related literature

For biological activity of *Saussurea involucrata*, see: Zheng *et al.* (1993 ▶); Gao *et al.* (2005 ▶); Tao *et al.*(2010 ▶); Ma *et al.* (2011 ▶); Jia *et al.* (2005 ▶); Liu *et al.* (1985 ▶). For related structures, see: Xiong *et al.* (2009 ▶); Vijayalakshmi *et al.* (1986 ▶); Paula *et al.* (2002 ▶).
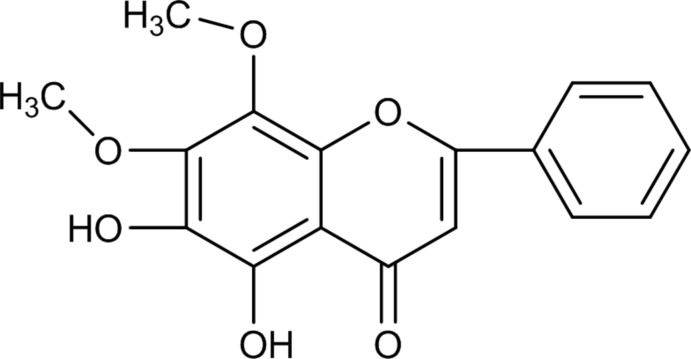


## Experimental

### 

#### Crystal data

C_17_H_14_O_6_
*M* *_r_* = 314.28
Triclinic, 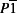

*a* = 7.953 (6) Å
*b* = 8.548 (6) Å
*c* = 10.951 (8) Å
α = 96.602 (8)°
β = 92.282 (8)°
γ = 100.279 (7)°
*V* = 726.3 (9) Å^3^
*Z* = 2
Mo *K*α radiation
μ = 0.11 mm^−1^
*T* = 295 K
0.21 × 0.16 × 0.09 mm

#### Data collection

Bruker APEXII CCD diffractometer
5252 measured reflections
3368 independent reflections
1786 reflections with *I* > 2σ(*I*)
*R*_int_ = 0.036

#### Refinement

*R*[*F* ^2^ > 2σ(*F* ^2^)] = 0.070
*wR*(*F* ^2^) = 0.236
*S* = 1.03
3368 reflections
212 parameters
H-atom parameters constrained
Δρ_max_ = 0.32 e Å^−3^
Δρ_min_ = −0.32 e Å^−3^

### 

Data collection: *APEX2* (Bruker, 2007 ▶); cell refinement: *SAINT* (Bruker, 2007 ▶); data reduction: *SAINT*; program(s) used to solve structure: *SHELXS97* (Sheldrick, 2008 ▶); program(s) used to refine structure: *SHELXL97* (Sheldrick, 2008 ▶); molecular graphics: *ORTEP-3* (Farrugia, 2012 ▶); software used to prepare material for publication: *SHELXTL* (Sheldrick, 2008 ▶) and *PLATON* (Spek, 2009 ▶).

## Supplementary Material

Crystal structure: contains datablock(s) global, I. DOI: 10.1107/S1600536813014451/rk2401sup1.cif

Structure factors: contains datablock(s) I. DOI: 10.1107/S1600536813014451/rk2401Isup2.hkl

Supplementary material file. DOI: 10.1107/S1600536813014451/rk2401Isup3.cml

Additional supplementary materials:  crystallographic information; 3D view; checkCIF report

## Figures and Tables

**Table 1:** Hydrogen-bond geometry (Å, °)

*D*—H⋯*A*	*D*—H	H⋯*A*	*D*⋯*A*	*D*—H⋯*A*
O4—H4⋯O3^i^	0.82	2.05	2.764 (4)	146
C12—H12⋯O6^ii^	0.93	2.58	3.234 (4)	128
O3—H3⋯O2	0.82	1.84	2.564 (3)	146
O4—H4⋯O3	0.82	2.34	2.767 (3)	113

Symmetry codes: (i) 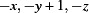; (ii) 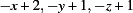.

**Fig. 1.**
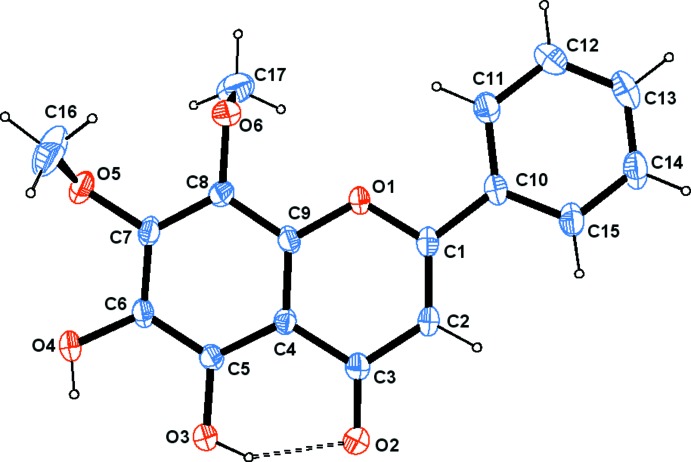
Molecular structure of the title compound with the atom numbering scheme. Displacement ellipsoids are drawn at the 30% probability level. H atoms are presented as a small spheres of arbitrary radius. Hydrogen bond is shown as dashed lines.

## supplementary crystallographic information

### Comment

*Saussurea involucrata* (Kar. et Kir) Sch.–Bip is one of the precious Tibetan herbs that have been used for a long period of time. Modern pharmacological studies have reported that the herb exhibit a wide range of bioactivitie, including antioxidation, anti–inflammatory, anti–fatigue, anti–hypoxia, anti–cancer and analgesic effects. (Zheng *et al.*, 1993; Gao *et al.*, 2005; Tao *et al.*, 2010; Ma *et al.*, 2011; Jia *et al.*, 2005; Liu *et al.*, 1985; Paula *et al.*, 2002).

Our chemical investigation of this herbs for components with anti–hypoxia activity resulted in the isolation of the title compound and crystal growth one, suitable for X–ray diffraction.

The molecular structure of title compound is almost planar; the dihedral angle between the benzopyran–4–one group and the attached phenyl group is 1.89 (6)° (Fig. 1).

In the crystal,the 5–hydroxy group forms a strong intramolecular hydrogen bond with the carbonyl group, resulting in a six–membered ring (Fig. 1). The centrosymmetrical dimers of title compound is linked by intermolecular O4—H4···O3^i^ hydrogen bonds. Non–classical C—H···O hydrogen bonds (C2—H2···O2^ii^, C15—H15···O2^ii^ and C14—H14···O4^iii^) and π–π interactions between molecule pairs (C3–C4–C5···C5^i^–C4^i^–C3^i^ = 3.37 (2)–3.39 (2)Å are found in the crystal structure. All of these interactions build up a three–dimensional network. Symmetry codes: (i) -*x*, -*y*+1, -*z*; (ii) 1-*x*, -*y*, -*z*; (iii) 1+*x*, 1+*y*, *z*.

### Experimental

The air–dried whole plants (10 kg) of *S. involucrate* were milled and extracted with 70% ethanol (150 L×3) for 2 h each time at 351 K. The resulting extract was concentrated to give ethanol extract (2.4 kg). The ethanol extract was suspended in water and extracted successively with equal volumes petroleum ether, *Et*O*Ac* and *n*–*Bu*OH. The petroleum ether extract (1.2 kg) was subjected to a silica gel column eluted with petroleum ether–*Et*O*Ac* (10:0 to 1:2, *v*/*v*) to afford five fractions. Fraction 5 was purified repeatedly over a Sephadex LH–20 column with a mixture of CHCl_3_–*Me*OH (1:1, *v*/*v*) to give title compound (500 mg). Single crystals suitable for X–ray diffraction analysis were obtained by slow evaporation from *Me*OH at room temperature.

^1^H–NMR (600 MHz, *DMS*–*d6*), *δ* (p.p.m.): 5.53 (1*H*, s, –OH), 12.27 (1*H*, s, –OH), 7.94 (2*H*, m), 7.57 (3*H*, m), 6.70 (1*H*, s), 4.16 (3*H*, s), 4.12 (3*H*, s). ^13^C–NMR (100 MHz, *DMS*–*d6*), *δ* (p.p.m.): 183.0, 164.2, 146.8, 143.1, 142.1, 133.3, 133.1, 132.0, 131.4, 129.1, 126.3, 106.8, 105.1, 62.2, 61.5.

### Refinement

In the structure the H atoms were positioned geometrically and refined with using a riding model: C—H = 0.96Å for methyl H; C—H = 0.97Å for methylene H and C—H = 0.93Å for aryl H with *U*_iso_(H) = 1.5*U*_eq_(C) for methyl H and *U*_iso_(H) = 1.2*U*_eq_(C) for other. Hydroxy H atoms were positioned with O—H = 0.82Å and *U*_iso_(H) = 1.5*U*_eq_(O).

### Crystal data

**Table:**

C_17_H_14_O_6_	*Z* = 2
*M**_r_* = 314.28	*F*(000) = 328
Triclinic, *P*1	*D*_x_ = 1.437 Mg m^−^^3^
*a* = 7.953 (6) Å	Mo *K*α radiation, λ = 0.71073 Å
*b* = 8.548 (6) Å	Cell parameters from 1607 reflections
*c* = 10.951 (8) Å	θ = 2.4–28.0°
α = 96.602 (8)°	µ = 0.11 mm^−^^1^
β = 92.282 (8)°	*T* = 295 K
γ = 100.279 (7)°	Block, orange
*V* = 726.3 (9) Å^3^	0.21 × 0.16 × 0.09 mm

### Data collection

**Table:**

Bruker APEXII CCD diffractometer	1786 reflections with *I* > 2σ(*I*)
Radiation source: fine-focus sealed tube	*R*_int_ = 0.036
Graphite monochromator	θ_max_ = 28.3°, θ_min_ = 2.4°
φ and ω scans	*h* = −9→10
5252 measured reflections	*k* = −10→11
3368 independent reflections	*l* = −14→14

### Refinement

**Table:**

Refinement on *F*^2^	Primary atom site location: structure-invariant direct methods
Least-squares matrix: full	Secondary atom site location: difference Fourier map
*R*[*F*^2^ > 2σ(*F*^2^)] = 0.070	Hydrogen site location: inferred from neighbouring sites
*wR*(*F*^2^) = 0.236	H-atom parameters constrained
*S* = 1.03	*w* = 1/[σ^2^(*F*_o_^2^) + (0.1286*P*)^2^] where *P* = (*F*_o_^2^ + 2*F*_c_^2^)/3
3368 reflections	(Δ/σ)_max_ < 0.001
212 parameters	Δρ_max_ = 0.32 e Å^−^^3^
0 restraints	Δρ_min_ = −0.32 e Å^−^^3^

### Special details

**Table:**

Geometry. All s.u.'s (except the s.u. in the dihedral angle between two l.s. planes) are estimated using the full covariance matrix. The cell s.u.'s are taken into account individually in the estimation of s.u.'s in distances, angles and torsion angles; correlations between s.u.'s in cell parameters are only used when they are defined by crystal symmetry. An approximate (isotropic) treatment of cell s.u.'s is used for estimating s.u.'s involving l.s. planes.
Refinement. Refinement of *F*^2^ against ALL reflections. The weighted *R*–factor *wR* and goodness of fit *S* are based on *F*^2^, conventional *R*–factors *R* are based on *F*, with *F* set to zero for negative *F*^2^. The threshold expression of *F*^2^ > σ(*F*^2^) is used only for calculating *R*–factors(gt) *etc*. and is not relevant to the choice of reflections for refinement. *R*–factors based on *F*^2^ are statistically about twice as large as those based on *F*, and *R*–factors based on ALL data will be even larger.

### Fractional atomic coordinates and isotropic or equivalent isotropic displacement parameters (Å^2^)

**Table:**

	*x*	*y*	*z*	*U*_iso_*/*U*_eq_	
O1	0.6956 (2)	0.4357 (2)	0.24860 (16)	0.0386 (5)	
O2	0.3542 (3)	0.1892 (2)	−0.02333 (18)	0.0489 (6)	
O3	0.1717 (2)	0.4078 (2)	0.00746 (18)	0.0450 (5)	
H3	0.1947	0.3212	−0.0196	0.067*	
O4	0.1217 (3)	0.6852 (2)	0.14665 (19)	0.0512 (6)	
H4	0.0594	0.6291	0.0909	0.077*	
O5	0.3574 (3)	0.8349 (2)	0.32644 (18)	0.0528 (6)	
O6	0.6457 (2)	0.7095 (2)	0.38048 (16)	0.0450 (5)	
C1	0.7247 (3)	0.2981 (3)	0.1857 (2)	0.0354 (6)	
C2	0.6135 (4)	0.2132 (3)	0.0965 (3)	0.0405 (7)	
H2	0.6368	0.1175	0.0576	0.049*	
C3	0.4604 (3)	0.2653 (3)	0.0593 (2)	0.0359 (6)	
C4	0.4325 (3)	0.4143 (3)	0.1259 (2)	0.0303 (6)	
C5	0.2887 (3)	0.4797 (3)	0.1000 (2)	0.0334 (6)	
C6	0.2615 (3)	0.6188 (3)	0.1678 (2)	0.0367 (6)	
C7	0.3817 (4)	0.6939 (3)	0.2618 (2)	0.0377 (7)	
C8	0.5273 (3)	0.6333 (3)	0.2885 (2)	0.0360 (6)	
C9	0.5500 (3)	0.4924 (3)	0.2197 (2)	0.0338 (6)	
C10	0.8873 (3)	0.2593 (3)	0.2294 (2)	0.0362 (6)	
C11	0.9879 (4)	0.3551 (4)	0.3266 (3)	0.0457 (7)	
H11	0.9524	0.4461	0.3637	0.055*	
C12	1.1388 (4)	0.3168 (4)	0.3683 (3)	0.0555 (9)	
H12	1.2037	0.3809	0.4340	0.067*	
C13	1.1942 (4)	0.1838 (4)	0.3130 (3)	0.0558 (9)	
H13	1.2969	0.1584	0.3407	0.067*	
C14	1.0974 (4)	0.0895 (4)	0.2172 (3)	0.0569 (9)	
H14	1.1342	−0.0010	0.1805	0.068*	
C15	0.9459 (4)	0.1264 (4)	0.1742 (3)	0.0492 (8)	
H15	0.8826	0.0620	0.1079	0.059*	
C16	0.2964 (6)	0.8185 (5)	0.4437 (4)	0.0927 (14)	
H16A	0.1833	0.7547	0.4353	0.139*	
H16B	0.2929	0.9224	0.4862	0.139*	
H16C	0.3712	0.7671	0.4897	0.139*	
C17	0.7790 (5)	0.8185 (4)	0.3370 (3)	0.0655 (10)	
H17A	0.8386	0.7615	0.2774	0.098*	
H17B	0.8575	0.8694	0.4048	0.098*	
H17C	0.7313	0.8982	0.2995	0.098*	

### Atomic displacement parameters (Å^2^)

**Table:**

	*U*^11^	*U*^22^	*U*^33^	*U*^12^	*U*^13^	*U*^23^
O1	0.0383 (11)	0.0392 (11)	0.0415 (10)	0.0216 (9)	−0.0029 (8)	−0.0015 (8)
O2	0.0437 (12)	0.0463 (12)	0.0544 (12)	0.0171 (9)	−0.0080 (9)	−0.0127 (9)
O3	0.0383 (12)	0.0452 (12)	0.0511 (12)	0.0182 (9)	−0.0083 (9)	−0.0080 (9)
O4	0.0429 (13)	0.0525 (13)	0.0612 (14)	0.0296 (10)	−0.0074 (10)	−0.0096 (10)
O5	0.0668 (15)	0.0453 (12)	0.0497 (12)	0.0305 (11)	0.0005 (10)	−0.0101 (9)
O6	0.0451 (12)	0.0519 (12)	0.0370 (11)	0.0143 (10)	−0.0040 (9)	−0.0044 (9)
C1	0.0384 (16)	0.0329 (14)	0.0393 (15)	0.0161 (12)	0.0063 (12)	0.0063 (11)
C2	0.0404 (17)	0.0367 (15)	0.0476 (16)	0.0201 (13)	0.0026 (13)	−0.0020 (12)
C3	0.0344 (15)	0.0381 (15)	0.0365 (14)	0.0121 (12)	0.0019 (11)	0.0009 (11)
C4	0.0321 (14)	0.0321 (14)	0.0290 (13)	0.0118 (11)	0.0047 (10)	0.0031 (10)
C5	0.0310 (15)	0.0388 (15)	0.0315 (13)	0.0112 (12)	0.0005 (11)	0.0027 (10)
C6	0.0340 (15)	0.0395 (15)	0.0406 (15)	0.0186 (12)	0.0018 (11)	0.0035 (11)
C7	0.0423 (17)	0.0371 (15)	0.0376 (14)	0.0198 (13)	0.0069 (12)	−0.0001 (11)
C8	0.0402 (16)	0.0409 (15)	0.0277 (13)	0.0140 (12)	0.0011 (11)	−0.0017 (10)
C9	0.0320 (15)	0.0403 (15)	0.0329 (14)	0.0166 (12)	0.0016 (11)	0.0052 (11)
C10	0.0338 (15)	0.0364 (15)	0.0433 (15)	0.0141 (12)	0.0069 (12)	0.0121 (11)
C11	0.0458 (18)	0.0488 (18)	0.0464 (16)	0.0202 (14)	0.0006 (13)	0.0051 (13)
C12	0.046 (2)	0.068 (2)	0.0549 (19)	0.0154 (17)	−0.0082 (15)	0.0124 (16)
C13	0.0407 (19)	0.065 (2)	0.071 (2)	0.0237 (17)	0.0030 (16)	0.0252 (18)
C14	0.048 (2)	0.0475 (19)	0.082 (2)	0.0252 (16)	0.0080 (17)	0.0113 (17)
C15	0.0446 (18)	0.0439 (17)	0.0628 (19)	0.0200 (14)	0.0016 (14)	0.0043 (14)
C16	0.134 (4)	0.090 (3)	0.066 (2)	0.055 (3)	0.041 (2)	−0.006 (2)
C17	0.064 (2)	0.063 (2)	0.060 (2)	−0.0004 (18)	0.0005 (17)	−0.0104 (16)

### Geometric parameters (Å, º)

**Table:**

O1—C1	1.355 (3)	C7—C8	1.384 (4)
O1—C9	1.372 (3)	C8—C9	1.389 (4)
O2—C3	1.250 (3)	C10—C11	1.393 (4)
O3—H3	0.8200	C10—C15	1.388 (4)
O3—C5	1.360 (3)	C11—H11	0.9300
O4—H4	0.8200	C11—C12	1.374 (4)
O4—C6	1.359 (3)	C12—H12	0.9300
O5—C7	1.375 (3)	C12—C13	1.377 (5)
O5—C16	1.404 (4)	C13—H13	0.9300
O6—C8	1.372 (3)	C13—C14	1.365 (4)
O6—C17	1.417 (4)	C14—H14	0.9300
C1—C2	1.343 (4)	C14—C15	1.378 (4)
C1—C10	1.467 (4)	C15—H15	0.9300
C2—H2	0.9300	C16—H16A	0.9600
C2—C3	1.429 (4)	C16—H16B	0.9600
C3—C4	1.450 (4)	C16—H16C	0.9600
C4—C5	1.393 (4)	C17—H17A	0.9600
C4—C9	1.385 (4)	C17—H17B	0.9600
C5—C6	1.384 (4)	C17—H17C	0.9600
C6—C7	1.391 (4)		
			
C1—O1—C9	119.6 (2)	C4—C9—C8	121.7 (2)
C5—O3—H3	109.5	C11—C10—C1	121.1 (2)
C6—O4—H4	109.5	C15—C10—C1	120.8 (3)
C7—O5—C16	114.1 (3)	C15—C10—C11	118.1 (3)
C8—O6—C17	112.7 (2)	C10—C11—H11	119.6
O1—C1—C10	111.1 (2)	C12—C11—C10	120.8 (3)
C2—C1—O1	121.9 (2)	C12—C11—H11	119.6
C2—C1—C10	127.0 (2)	C11—C12—H12	119.9
C1—C2—H2	119.0	C11—C12—C13	120.2 (3)
C1—C2—C3	122.1 (2)	C13—C12—H12	119.9
C3—C2—H2	119.0	C12—C13—H13	120.2
O2—C3—C2	123.7 (2)	C14—C13—C12	119.5 (3)
O2—C3—C4	120.9 (2)	C14—C13—H13	120.2
C2—C3—C4	115.4 (2)	C13—C14—H14	119.5
C5—C4—C3	121.9 (2)	C13—C14—C15	121.0 (3)
C9—C4—C3	119.3 (2)	C15—C14—H14	119.5
C9—C4—C5	118.8 (2)	C10—C15—H15	119.8
O3—C5—C4	120.6 (2)	C14—C15—C10	120.3 (3)
O3—C5—C6	118.5 (2)	C14—C15—H15	119.8
C6—C5—C4	120.9 (2)	O5—C16—H16A	109.5
O4—C6—C5	122.7 (2)	O5—C16—H16B	109.5
O4—C6—C7	118.5 (2)	O5—C16—H16C	109.5
C5—C6—C7	118.8 (2)	H16A—C16—H16B	109.5
O5—C7—C6	118.7 (2)	H16A—C16—H16C	109.5
O5—C7—C8	119.5 (2)	H16B—C16—H16C	109.5
C8—C7—C6	121.7 (2)	O6—C17—H17A	109.5
O6—C8—C7	121.1 (2)	O6—C17—H17B	109.5
O6—C8—C9	120.8 (2)	O6—C17—H17C	109.5
C7—C8—C9	118.1 (2)	H17A—C17—H17B	109.5
O1—C9—C4	121.7 (2)	H17A—C17—H17C	109.5
O1—C9—C8	116.6 (2)	H17B—C17—H17C	109.5
			
O1—C1—C2—C3	2.1 (4)	C3—C4—C9—C8	−178.3 (2)
O1—C1—C10—C11	2.1 (4)	C4—C5—C6—O4	−179.0 (2)
O1—C1—C10—C15	−177.4 (3)	C4—C5—C6—C7	0.8 (4)
O2—C3—C4—C5	−0.7 (4)	C5—C4—C9—O1	−179.0 (2)
O2—C3—C4—C9	177.8 (2)	C5—C4—C9—C8	0.3 (4)
O3—C5—C6—O4	0.9 (4)	C5—C6—C7—O5	177.9 (2)
O3—C5—C6—C7	−179.3 (2)	C5—C6—C7—C8	0.3 (4)
O4—C6—C7—O5	−2.3 (4)	C6—C7—C8—O6	179.2 (2)
O4—C6—C7—C8	−179.9 (3)	C6—C7—C8—C9	−1.0 (4)
O5—C7—C8—O6	1.6 (4)	C7—C8—C9—O1	−180.0 (2)
O5—C7—C8—C9	−178.6 (2)	C7—C8—C9—C4	0.7 (4)
O6—C8—C9—O1	−0.2 (4)	C9—O1—C1—C2	−1.1 (4)
O6—C8—C9—C4	−179.5 (2)	C9—O1—C1—C10	179.3 (2)
C1—O1—C9—C4	−1.3 (4)	C9—C4—C5—O3	179.1 (2)
C1—O1—C9—C8	179.4 (2)	C9—C4—C5—C6	−1.1 (4)
C1—C2—C3—O2	180.0 (3)	C10—C1—C2—C3	−178.3 (2)
C1—C2—C3—C4	−0.9 (4)	C10—C11—C12—C13	1.0 (5)
C1—C10—C11—C12	179.0 (3)	C11—C10—C15—C14	1.5 (5)
C1—C10—C15—C14	−178.9 (3)	C11—C12—C13—C14	−0.6 (5)
C2—C1—C10—C11	−177.5 (3)	C12—C13—C14—C15	0.7 (5)
C2—C1—C10—C15	3.0 (4)	C13—C14—C15—C10	−1.2 (5)
C2—C3—C4—C5	−179.9 (2)	C15—C10—C11—C12	−1.5 (5)
C2—C3—C4—C9	−1.3 (4)	C16—O5—C7—C6	103.2 (4)
C3—C4—C5—O3	−2.4 (4)	C16—O5—C7—C8	−79.2 (4)
C3—C4—C5—C6	177.5 (2)	C17—O6—C8—C7	−92.6 (3)
C3—C4—C9—O1	2.4 (4)	C17—O6—C8—C9	87.6 (3)

### Hydrogen-bond geometry (Å, º)

**Table:**

*D*—H···*A*	*D*—H	H···*A*	*D*···*A*	*D*—H···*A*
O4—H4···O3^i^	0.82	2.05	2.764 (4)	146
C12—H12···O6^ii^	0.93	2.58	3.234 (4)	128
O3—H3···O2	0.82	1.84	2.564 (3)	146
O4—H4···O3	0.82	2.34	2.767 (3)	113
C11—H11···O1	0.93	2.34	2.675 (4)	101

Symmetry codes: (i) −*x*, −*y*+1, −*z*; (ii) −*x*+2, −*y*+1, −*z*+1.
